# Current guidelines for *BRCA* testing of breast cancer patients are insufficient to detect all mutation carriers

**DOI:** 10.1186/s12885-017-3422-2

**Published:** 2017-06-21

**Authors:** Eli Marie Grindedal, Cecilie Heramb, Inga Karsrud, Sarah Louise Ariansen, Lovise Mæhle, Dag Erik Undlien, Jan Norum, Ellen Schlichting

**Affiliations:** 1 Department of Medical Genetics, Oslo University Hospital, Oslo, Norway; 2 Norwegian National Advisory Unit on Women’s Health, Oslo University Hospital, Rikshospitalet, Oslo, Norway; 3 Department of Medical Genetics, Oslo University Hospital and University of Oslo, Oslo, Norway; 4 Section for Breast- and Endocrine Surgery, Department of Cancer, Oslo University Hospital, Oslo, Norway; 5 Northern Norway Regional Health Authority trust, Bodø, Norway; 6 Medical Imaging Research Group, Department of Clinical Medicine, UiT-The Arctic University of Norway, Tromsø, Norway

**Keywords:** Breast cancer, BRCA mutation, Genetic testing, Norway

## Abstract

**Background:**

Identification of *BRCA* mutations in breast cancer (BC) patients influences treatment and survival and may be of importance for their relatives. Testing is often restricted to women fulfilling high-risk criteria. However, there is limited knowledge of the sensitivity of such a strategy, and of the clinical aspects of BC caused by *BRCA* mutations in less selected BC cohorts. The aim of this report was to address these issues by evaluating the results of *BRCA* testing of BC patients in South-Eastern Norway.

**Methods:**

1371 newly diagnosed BC patients were tested with sequencing and Multi Ligation Probe Amplification (MLPA). Prevalence of mutations was calculated, and BC characteristics among carriers and non-carriers compared. Sensitivity and specificity of common guidelines for *BRCA* testing to identify carriers was analyzed. Number of identified female mutation positive relatives was evaluated.

**Results:**

A pathogenic *BRCA* mutation was identified in 3.1%. Carriers differed from non-carriers in terms of age at diagnosis, family history, grade, ER/PR-status, triple negativity (TNBC) and Ki67, but not in HER2 and TNM status. One mutation positive female relative was identified per mutation positive BC patient. Using age of onset below 40 or TNBC as criteria for testing identified 32-34% of carriers. Common guidelines for testing identified 45-90%, and testing all below 60 years identified 90%. Thirty-seven percent of carriers had a family history of cancer that would have qualified for predictive *BRCA* testing. A Variant of Uncertain Significance (VUS) was identified in 4.9%.

**Conclusions:**

Mutation positive BC patients differed as a group from mutation negative. However, the commonly used guidelines for testing were insufficient to detect all mutation carriers in the BC cohort. Thirty-seven percent had a family history of cancer that would have qualified for predictive testing before they were diagnosed with BC. Based on our combined observations, we suggest it is time to discuss whether all BC patients should be offered *BRCA* testing, both to optimize treatment and improve survival for these women, but also to enable identification of healthy mutation carriers within their families. Health services need to be aware of referral possibility for healthy women with cancer in their family.

**Electronic supplementary material:**

The online version of this article (doi:10.1186/s12885-017-3422-2) contains supplementary material, which is available to authorized users.

## Background

Germline mutations in the *BRCA1* and *BRCA2* genes are associated with a high lifetime risk of breast and ovarian cancer [1, 2]. Knowledge of one’s *BRCA* status is of importance for healthy women as cancer may be prevented through risk-reducing mastectomy and salpingo-oophorectomy [3–5]. Identification of a pathogenic *BRCA* mutation in a woman diagnosed with breast cancer (BC) may influence treatment and prognosis of her current cancer but also enable prevention of future cancers [6–12]. Consequently, surgeons and oncologists more and more frequently want to offer genetic testing at time of diagnosis.

Because of the high costs associated with genetic analyses, *BRCA1/2*- testing has traditionally been restricted to BC patients having an a priori high risk of being a carrier. These factors include young age at diagnosis (below 45 years), triple-negative breast cancer (TNBC) or a family history of breast- and/or ovarian cancer [13–22]. The American Society of Clinical Oncology (ASCO), The National Comprehensive Cancer Network (NCCN) in the US and the Norwegian Breast Cancer Group (NBCG) all have guidelines for *BRCA* testing of BC patients based on these risk factors (Additional file 1: Figure S1), and according to The National Institute for Health and Care Excellence (NICE) in the UK, *BRCA* testing should be offered to BC patients with a probability of having a mutation is 10% or more [23–26]. There are also corresponding guidelines for predictive testing of healthy women.

During the recent years, the cost of genetic testing has decreased due to the advent of new and more efficient DNA-sequencing technologies. Consequently, BC patients are now often offered multi gene panel testing. These panels include *BRCA1/2* and the other high risk breast cancer genes *TP53* and *PTEN*, but also genes with more moderate cancer risk and genes whose clinical significance is still not resolved [27, 28]. Testing is nevertheless still mostly restricted to patients fulfilling certain high risk criteria for being mutation carriers, and few studies have described *BRCA* testing of unselected groups of BC patients [29–35]. To our knowledge, only two studies have performed testing with sequencing and Multi-Ligation Probe Amplification (MLPA) of all patients included [30, 35]. Knowledge of the clinical characteristics of BC caused by *BRCA* mutations in unselected BC cohorts is therefore limited. Moreover, there is also limited information about the sensitivity and specificity of current guidelines for *BRCA* testing to identify carriers in cohorts not selected for high risk factors. With the ongoing changes in opportunities for genetic testing we believe it is necessary to assess whether the current strategies for *BRCA* testing are sufficient to enable mutation positive women to benefit from the potential of both cancer cure and prevention that lies within such testing. Observations from *BRCA* testing of less selected groups of BC patients are necessary for this evaluation.

The NBCG guidelines used in Norway are regularly revised. Because it became clear that identification of a *BRCA* mutation could have implications for treatment, a subjective criteria was introduced a few years ago. If the treating physician considered the test result to be of importance for treatment decisions, testing could be offered even in the absence of other high risk factors such as young age or family clustering. As a consequence, testing could be offered also to BC patients with an a priori low risk of being carriers. Due to this change in practice we have been able to compare the sensitivity of previous and present national and international guidelines for *BRCA* testing in BC patients without the selection bias described.

This report summarizes the results of *BRCA* testing in South-Eastern Norway according to these revised Norwegian guidelines from 1st of January 2014 to 31st of August 2015. The study had three specific aims: Firstly, it was to calculate the prevalence of *BRCA* mutations in this cohort of BC patients that as a whole had an a priori low risk of being mutation carriers, describe the spectrum of mutations, and the number of mutation positive female relatives identified. Secondly, we wanted to describe and compare clinicopathological features of BC among carriers and non-carriers. The third aim was to calculate the sensitivity and specificity of different guidelines used for diagnostic testing [23–26], and also to evaluate how many mutation carriers that had a family history of cancer that qualified for predictive testing before they were diagnosed with BC [26].

## Methods

### Patients

During the study period, a total of 1371 BC patients were tested. Two cohorts of patients are described in this report: Cohort 1: Patients tested at The Breast Cancer Surgery Unit, Department of Oncology, Oslo University Hospital, Ullevål (OUH-U), and Cohort 2: Patients tested at the other hospitals in the health administrative area of South Eastern Norway called South-Eastern Norway Regional Health Authority trust. This cohort is referred to as SERHA.

#### OUH-U (cohort 1)

This is the largest unit treating BC patients in Norway. Six hundred and seven patients underwent BC surgery, and 440 (72.5%) of them were tested. Two of these were men. A quality of care database was established at the unit to evaluate the practice of *BRCA* testing among this group of patients. Information on age of onset, receptor status, grade, stage, nodal involvement, Ki67 and family history was accessed from the Electronic Patient Record (EPR) system (DIPS®) and registered in the quality database. Family history was taken by the doctor admitting the patient to the hospital according to ordinary routines. No standardized or quality assured methods were used. The information on family history recorded in the patient record of both carriers and non-carriers was evaluated and scored according to the old diagnostic and predictive test criteria of NBCG [26]. No information on size (number of family members) of the families was recorded. One hundred and sixty-seven patients were not tested. Of these, 96 either directly declined testing or wanted to think about it. For the remaining 71, there was no record in the hospital’s EPR system on whether testing was offered or not.

#### SERHA (cohort 2)

We do not have the exact number of all BC patients undergoing treatment at these hospitals the other hospitals in the health region during the study period, but based on numbers from the Norwegian Breast Cancer Registry (NBCR) at the Cancer Registry of Norway (CRN) we estimated that the number was around 2400 [36]. Nine hundred and thirty-one (39.0%) were tested. Information on age of onset, receptor status and family history was registered on all carriers in the EPR at the Department of Medical Genetics (DMG) OUH. No information was collected on mutation negatives in this cohort.

### Genetic testing

Genomic DNA was purified from EDTA-anticoagulated blood using the QiaSymphony instrument (Qiagen, Hilden, Germany). All 23 coding exons of *BRCA1* (exons 2 to 24) and 26 coding exons of *BRCA2* (exons 2 to 27), were amplified, the primers were designed to cover all coding exons and adjacent 20–base pair introns. The amplified DNA fragments were sequenced using the BigDyeTerminator Cycle Sequencing kit on an ABI 3730 DNA Analyzer (Applied Biosystems, Foster City, CA). All sequences were compared with the *BRCA1* (NM_007294.3) and *BRCA2* (NM_000059.3) reference sequences for variant detection. In addition, MLPA (P002 *BRCA1* and P045 *BRCA2* MLPA probe mixes; MRC-Holland, Amsterdam, The Netherlands) was performed to identify deletions and insertions.

Results were interpreted and reported following the recommendations of the American College of Medical Genetics [37], using the five-class system. Patients with a variant class 4 or 5, patients with a normal test, but with a young age of onset and/or a family history of BC, and patients with a Variant of Uncertain Significance (VUS) were all referred to genetic counseling at DMG OUH. Here, they received genetic counseling, a detailed family history was obtained and relevant diagnoses in relatives confirmed. A quality of care database was established at DMG OUH and all BC patients with a pathogenic *BRCA* mutation and their relatives who were tested for the mutation were registered here. Both male and female relatives of the mutation positive BC patients were offered testing for the mutation in question. Testing was offered not only to first degree relatives, but to all blood relatives who were referred to DMG OUH.

### Statistics

Mutation carriers from both cohorts were scored according to the ASCO, NCCN, NICE and NBCG guidelines [23–26]. Carriers were scored according to the NBCG criteria as they were before the revision that opened for testing based on implication for treatment decisions. In the remainder of the article these will be referred to as the “old NBCG criteria”. To score patients according to the NICE guidelines, the BOADICEA Web Application (BWA v3) [38] was used to calculate risk of carrying a *BRCA* mutation. Sensitivities of criteria to identify carriers were calculated excluding the patients with a known family mutation.

Tests for trends were performed to compare the differences in BC characteristics between mutation carriers and non-carriers. Separate analyses were done to compare tested and non-tested in order to illustrate potential bias in the group that was not tested. Mutation positives in Cohort 1 and 2 were compared to investigate how similar the two cohorts were. Pearson’s Chi square and one-way ANOVA were used to compare categorical variables (ER, PR, HER2 status, grade, stage, nodal involvement, family history, Ki67 ≥ 30%) while independent t-tests were used to compare continuous variables (age, mean Ki67). In all analyses, *p*-values less than 0.05 were considered statistically significant. All statistical analyses were performed using SPSS version 21.0. When missing values were observed, this case was omitted in the analysis of this variable.

## Results

### Identified mutation carriers, spectrum and frequency of mutations

A pathogenic mutation in *BRCA1/2* was identified in 42 of the 1371 (3.1%) BC patients. Thirteen mutation carriers were identified in Cohort 1 (13/400 = 3.0%), and 29 in Cohort 2 (29/931 = 3.1%). All mutation carriers were women. Twenty-eight (2.0%) had a mutation in *BRCA1* and 14 (1.0%) in *BRCA2*. Median and mean age at diagnosis was 45 years (range 26-77 years) and 46.1 years (46.3 years for *BRCA1* and 45.6 years for *BRCA2*) respectively. Four of the 42 women belonged to families where a *BRCA* mutation already had been detected, but had not sought predictive genetic testing. Four of the mutation carriers were detected through MLPA (dup exon 3-16, dup exon 13 and del exon 22 in *BRCA1* and dup exon 20 in *BRCA2*), and the remaining carriers with sequencing. A VUS was identified in 67 (4.9%) patients.

When considering only those with Norwegian ancestry, we revealed that 13/29 (44.8%) had one of the known Norwegian founder mutations [39]. Eleven of 29 (37.9%) had a mutation previously found in 1- 9 families at DMG (unpublished data), and 5/29 (17.2%) had a mutation not previously observed in Norway. One of these was *BRCA2* c.614delG. Two patients carried this mutation and were related. Of the 13 mutation carriers that were not of Norwegian ancestry, three were from Poland and two from Morocco. The following nationalities were represented with one carrier each: Canadian, Swedish, Iraqi, Latvian, Indian, Turkish, and Greek. Three different *BRCA2* mutations were identified in the three BC patients from Poland. None of them were among the mutations known to be frequent in the Polish population [40–42], and only one of them had been reported previously (c.9403delC) [42]. The other two (c.4797_4797delCAAT and c.7024C > T) were not found to be reported previously in the Polish population. Mutation, age of onset, nationality, fulfilling criteria for predictive testing or not and clinicopathological aspects of tumors among mutation carriers is presented in Table 1. Age at diagnosis is given in age ranges to prevent disclosing patient information.

**Table 1:** Identified *BRCA1/2* carriers

Gene	Cohort	Mutation	Effect	Age at diagnosis	Norwegian ancestry	Qualifying for predictive testing	Triple negative disease
*BRCA1*	1	c.2019delA	Frameshift	20-29	No	No	Yes
	1	c.1016dupA^a^	Frameshift	30-39	Yes	Yes	Yes
	1	del exon 3-16	Deletion	30-39	No	No	No
	1	c.3228_3229delAG^a*^	Frameshift	30-39	Yes	No	No
	1	c.3178G > T^a^	Nonsense	40-49	Yes	No	No
	1	c.3084_3094delTAATAACATTA^a^	Frameshift	40-49	Yes	No	Yes
	1	c.3228_3229delAG^a^	Frameshift	40-49	Yes	No	Yes
	1	c.5047G > T^b^	Nonsense	40-49	Yes	Yes	Yes
	1	c.3607C > T^b^	Nonsense	50-59	Yes	Yes	No
	1	c.4484G > A^c^	Missense. Leads to skipping of exon 14	50-59	Yes	No	No
	2	c.5407-2A > G^c^	Frameshift, skipping of exon 23	60-69	Yes	No	No
	2	c.1072delC^b^	Frameshift	60-69	Yes	No	No
	2	c.1556delA^a^	Frameshift	40-49	Yes	Yes	No
	2	c.5153G > C^b^	Missense	40-49	Yes	Yes	No
	2	c.3756_3759delGTCT	Frameshift	40-49	No	No	No
	2	del exon 22	Frameshift	40-49	No	No	Yes
	2	c.5309G > T	Missense	30-39	No	Yes	Yes
	2	c.697delGT^a^	Frameshift	70-	Yes	Yes	Yes
	2	c.3228_3229delAG^a^	Frameshift	50-59	Yes	Yes	Yes
	2	c.445G > T	Nonsense	40-49	No	Yes	Yes
	2	c.1016dupA^a^	Frameshift	50-59	Yes	No	Yes
	2	c.5266dupC	Frameshift	30-39	No	Yes	No
	2	c.2989_29x0dup^b^	Frameshift	50-59	Yes	No	No
	2	c.1016dupA^a^	Frameshift	30-39	Yes	No	No
	2	c.1556delA^a^	Frameshift	50-59	Yes	Yes	No
	2	dup exon 13^b^	Frameshift	50-59	Yes	No	No
	2	c.5309G > T	Missense	30-39	No	No	Yes
	2	c.5503C > T	Nonsense	50-59	No	Yes	Yes
*BRCA2*							
	1	c.4710delA	Frameshift	30-39	No	No	No
	1	c.3847_3848delGT^a^	Frameshift	40-49	Yes	No	No
	1	c.614delG^c^	Frameshift	50-59	Yes	Yes	No
	2	c.4936_4939delGAAA^b^	Frameshift	40-49	Yes	No	No
	2	c.3847delGT^a^	Frameshift	40-49	Yes	No	No
	2	c.9403delC	Frameshift	40-49	No	No	No
	2	c.5722delCT^b^	Frameshift	40-49	Yes	No	No
	2	c.6059_6062delAACA^b^	Frameshift	30-39	Yes	No	No
	2	c.5722delCT^b^	Frameshift	50-59	Yes	Yes	No
	2	c.4794_4797delCAAT	Frameshift	40-49	No	Yes	No
	2	c.614delG^c^	Frameshift	30-39	Yes	Yes	No
	2	c.7024C > T	Nonsense	30-39	No	Yes	No
	2	c.9699_9702delTATG^c^	Frameshift	70-	Yes	No	No
	2	dup exon 20^b^	Frameshift	40-49	Yes	Yes	Yes

1: Tested at Oslo University Hospital Ullevål (OUH-U)

2: Tested at other hospitals in South-Eastern Norway Regional Health Authority trust’s coverage area (SERHA)

^a^Common Norwegian founder mutation^38^

^b^Identified in 1-9 families at Department of Medical Genetics (DMG), OUH (unpublished data)

^c^Not identified previously at DMG, OUH

*BC* Breast cancer

*OC* Ovarian cancer

As of August 2016, 67 female and 19 male relatives of the 42 mutation positive BC patients have been tested for the mutation identified in their family. Forty female relatives have tested positive for the mutation identified in their family. Five of the 42 BC patients had no adult female relatives living in Norway. Excluding these 5, 40/37 = 1.1 female mutation positive female relative has so far been identified per mutation positive BC patient. This number is likely to increase as more relatives are informed and tested. The mean age in this group of carriers was 46.7 years (range 20-84). All were offered annual MRI and mammography from the age of 25, and they were given the opportunity of choosing risk-reducing surgery. Seven of the relatives had already had cancer before the mutation was identified in their relative. Five of these had had BC and two OC. In addition, after being tested for the mutation in their family, one woman has been diagnosed with BC at first MRI and one has been diagnosed with OC with FIGO stage 1B when undergoing prophylactic salpingo-oophorectomy. In addition to those who have been tested, 37 female relatives (first degree or second degree through a man) aged above 18 years and 17 below 18 years have been identified, but they have not yet been referred for testing.

### Comparison of clinicopathological characteristics of tumors in mutation positive and mutation negative from the OUH-U cohort

No information was collected about the mutation negative BC patients in Cohort 2 from SERHA. A detailed comparison of the clinicopathological characteristics of tumors in mutation positive and mutation negative was therefore only possible to perform in cohort 1 from OUH-U. The results are presented in Table 2. Compared to the mutation negative, mutation positive women were younger (*p* < 0.001), had tumors of higher grade (*p* = 0.001), higher Ki67 (*p* < 0.001 (comparing mean) and *p* = 0.004 (comparing number with <30% activity) and more of them had TNBC (*p* < 0.001). In addition, more mutation carriers had family histories of breast and/or ovarian cancer compared to BC patients without mutation (*p* = 0.035). No significant difference was observed in TNM- status (*p* = 0.396) and HER2-profile (*p* = 0.84). In cohort 1 from OUH-U, 167 patients were not tested. They had a higher age at diagnosis (*p* < 0.001), a lower Ki67 score (*p* < 0.05) and a lower proportion fulfilled the old NBCG criteria (*p* < 0.05) compared to the patients tested (Additional file 2: Table S1).

**Table 2:** Comparison of clinical and pathological characteristics of carriers and non-carriers tested at Oslo University Hospital Ullevål (OUH-U)

	*BRCA 1/2* carriers (*n* = 13)	Non-carriers (*n* = 427)	*p*-values
Below 40 years	5 (38.5%)	24 (5.6%)	
Below 50 years	10 (76.9%)	106 (24.8%)	
Below 60 years	13 (100%)	222 (52.0%)	
Age at diagnosis			
Mean (95% CI)	42 (36.1-47.9)	57.9 (56.8-59.1)	<0.001
Median (range)	43 (26-58)	58 (23-93)	
Predictive test criteria fulfilled	4 (30.8%)	49 (11.5%)	0.035
Stringent NBCG criteria fulfilled	12 (92.3%)	130 (30.4%)	<0.001
TNM	(*n* = 13)	(*n* = 422)	
T1	5 (38.5%)	261 (61.8%)	
T2	5 (38.5%)	121 (28.7%)	0.396
T3	2 (15.3%)	26 (6.2%)	
T4	1 (7.7%)	13 (3.1%)	
N0	8 (61.5%)	295 (72.0%)	
N1	2(15.4%)	82 (19.4%)	0.078
N2	3(23.1%)	24 (5.7%)	
N3	0	12 (2.8%)	
Distant metastasis	0	2 (0.5%)	0.76
Grade	(*n* = 13)	(*n* = 419)	
1	0	101 (24.1%)	0.001
2	4 (30.8%)	211 (50.4%)	
3	9 (69.2%)	107 (25.5%)	
Estrogen receptor status	(*n* = 13)	(*n* = 423)	
Positive	7 (53.8%)	371 (87.7%)	<0.001
Negative	6 (46.2%)	52 (12.3%)	
Progesterone receptor status	(*n* = 13)	(*n* = 422)	0.045
Positive	5 (38.5%)	276 (65.4%)	
Negative	8 (61.5%)	146 (34.6%)	
HER2 status	(*n* = 13)	(*n* = 423)	
Positive	2 (15.4%)	57 (13.5%)	0.84
Negative	11 (84.6%)	366 (86.5%)	
Triple negative breast cancer	(*n* = 13)	(*n* = 422)	
	5 (38.5%)	30 (7.1%)	<0.001
Ki67	(*n* = 13)	(*n* = 412)	
Mean (95% CI)	59 (45.3-72.8)	31.3 (29-33)	<0.001
< 30% activity	11 (84.6%)	182 (44.1%)	0.004

*NBCG* Norwegian Breast Cancer Group

*TNM* Scoring of tumors according to the TNM Classification of Malignant Tumors

*T* Size of original tumor

*N* Involvement of regional lymph nodes

*M* Distant metastasis

To indirectly assess whether the two cohorts were similar in terms of risk distribution, we compared mutation positive patients in the two groups in terms of age at diagnosis, receptor status and family history of cancer. There was a tendency towards a higher mean age of onset in Cohort 2 compared to Cohort 1 (48 vs 42, *p* = 0.09). There was no significant difference in terms of TNBC and whether or not they fulfilled the diagnostic NBCG criteria (Additional file 3: Table S2).

### Sensitivity and specificity of criteria for genetic testing

The old NBCG, the NCCN, and ASCO guidelines had a sensitivity ranging from 84.2% to 89.5%. The NICE guidelines had the lowest sensitivity, and would have identified only 44.7% of the mutation positive women. Testing only women below 40 years or only those with TNBC would have identified 31.6% and 34.2% of the mutation carriers. Testing all BC patients below 60 years would have identified 89.5%. Almost 40% of the BC patients found to carry a *BRCA* mutation had a family history of cancer that fulfilled the NBCG criteria for predictive *BRCA* testing, before they were diagnosed with BC themselves. See Table 3 for details.

**Table 3:** Sensitivity of criteria for testing to identify *BRCA1/2* carriers

Test criteria	*BRCA1/2* mutation carriers (*n* = 38^a^)
BC <40 years	12 (31.6%)
BC <50 years	28 (73.7%)
BC <60 years	34 (89.5%)
TNBC	13 (34.2%)
Fulfilling stringent NBCG criteria for testing	32 (84.2%)
Fulfilling ASCO guidelines for testing	34 (89.5%)
Fulfilling NICE guidelines for testing	17 (44.7%)
Fulfilling NCCN criteria for testing	32 (88.9%)
Family history fulfilling NBCG criteria for predictive testing before index person contracted BC	14 (36.8%)

^a^The four women belonging to families where a mutation had already been identified were excluded from this analysis

*BC* Breast cancer

*TNBC* Triple Negative Breast Cancer

*NBCG* Norwegian Breast Cancer Group

*ASCO* American Society of Clinical Oncology

*NICE* National Institute for Health and Care Excellence

*NCCN* The National Comprehensive Cancer Network

The specificity of the different criteria for testing was calculated for Cohort 1 from OUH, and is presented in Table 4. The highest specificity was found for the high-risk criteria separately of each other. Breast cancer <40 years of age and TNBC both had a specificity of 94%. The specificity of fulfilling the NBCG criteria was 70%, while having breast cancer below 60 years of age had a specificity of 48%. Mutation frequency and number needed to test (NNT) to identify one mutation carrier depending on different test criteria are shown in Table 5. Testing all BC patients below 60 years would give mutation frequency of 5.5% and by using this criteria, 18 BC patients had to be tested to identify one carrier.

**Table 4:** Specificity of criteria for *BRCA1/2* testing

Test criteria	Specificity^a^
BC < 40 years	(403/427) 94.4%
BC < 50 years	(321/427) 75.2%
BC < 60 years	(205/427) 48%
TNBC	(397/422) 94.1%
Fulfilling NBCG criteria for diagnostic testing	(297/427) 69.5%
Fulfilling NBCG criteria for predictive testing	(378/427) 89%

^a^Specificity is calculated only for Cohort 1, OUH-U

*BC* Breast cancer

*TNBC* Triple negative breast cancer

*NBCG* Norwegian breast cancer group

**Table 5:** Number needed to test to identify one mutation carrier according to test criteria

Test criteria	Mutation frequency	Number needed to test (NNT) to identify one mutation carrier
BC < 50 years	10/116 = 8.6%	12
BC < 60 years	13/235 = 5.5%	18
TNBC	5/35 = 14%	7
NBCG criteria	12/147 = 8.2%	12

## Discussion

We have reported the results of diagnostic *BRCA* testing of women diagnosed with BC in the South-Eastern part of Norway according to the NBCG guidelines. These guidelines opened up for testing independently of the common high risk factors i.e. also when the treating physician considered the test result to be of importance for treatment decisions. To our knowledge, this is therefore the largest and least selected series reported where BC patients were tested with both sequencing and MLPA of both genes, and it does not have the selection bias arising when only high-risk patients are tested.

We identified a mutation in 3.1% of BC patients. In a recent study from the Western region of Norway, 405 BC patients were tested for 30 specific *BRCA1*/*2* mutations and with MLPA [32]. Sequencing was performed on 94 of these. A mutation was found in only 1.7% of participants. Both studies are small and consequently they do have limitations. However, the observed difference may at least partly be explained by the fact that all patients in our study were tested with sequencing and MLPA and not for selected mutations only. In our study, 16 out of 29 (55%) women with Norwegian ancestry did not have any of the 10 most common Norwegian founder mutations [39], and five (17%) had a mutation that had not been previously observed in our population. In comparison, in 2007 the 10 founder mutations accounted for about two-thirds of all detected mutation carriers at our department [39]. This reflects that in 2007 most patients were tested for a limited number of mutations, whereas today sequencing and MLPA is offered to all who qualify for testing in our health region. Our findings also illustrate that there are mutations within our population that are and may remain rare. By testing only for frequently observed mutations in the Norwegian population, a substantial number of mutation positive women with a pathogenic *BRCA* mutation will not be found.

A VUS was identified in 4.9% of the tested patients. Our numbers are comparable to what others have revealed [43]. Studies have reported that physicians, with limited formal training in genetics, may misinterpret VUS results [44–46]. This was dealt with in the current study as all patients with a VUS were referred to genetic counseling. There is a worry that information about a VUS may have a negative psychological impact on the patient [47]. However, studies have also demonstrated that it is interpreted as more similar to a test result where no pathogenic variant has been detected than to a result with an identified pathogenic variant [46]. Addressing the issue of patients’ interpretation of risk and possible psychological impact was beyond the scope of this study, but should be closer evaluated in future studies. By offering testing only for a set of already known and described mutations one would avoid the challenges associated with identifying VUS. We have however described that a substantial number of mutation carriers will be missed by testing only for known mutations. It is our opinion that the benefits associated with identifying all carriers (and the corresponding risk associated with not identifying a mutation carrier) outweigh the current challenges associated with identifying VUS. One may also hypothesize that the frequency of VUS may decrease in the future as more people are undergoing testing.

By comparing carriers and non-carriers tested at OUH-U we observed that even though testing was offered broadly, mutation positive women still differed from mutation negative in terms of the known high risk aspects for being carriers: age of onset, triple negativity and family history. We found no difference in HER2-status between the two groups, and these findings are in accordance with a recent study where HER2-status was not found to be a reliable predictor of *BRCA*-status [48]. Mutation carriers had a higher score for Ki67 than mutation negatives, and this has also been described in a few studies [49, 50]. The observed differences between the two groups are also illustrated by the fact that each of the test criteria has a high specificity (see Table 4).

Whereas the mutation positive differed as a group from mutation negative, selecting patients for testing based on the known high risk factors will identify carriers with varying sensitivity (see Table 3). Testing only those with BC below 40 years or TNBC identified 31.6% and 34.2% of carriers respectively, and less than 50% of carriers qualified for testing according to the NICE guidelines. By use of the comprehensive ASCO, NCCN and old NBCG criteria, where the different single characteristics are combined in order to increase sensitivity, between 84.2 and 89.5% would be identified. NBCG has recently suggested that testing should be offered to women with TNBC under the age of 60 [26]. By adding this aspect to the original stringent criteria, 34/38 (89.5%) would have been identified. In a recent study where 488 women with BC were tested for mutations in 25 cancer susceptibility genes, Tung et al. found that all *BRCA*-mutation carriers fulfilled the NCCN guidelines [35]. We do not know whether the difference in observed sensitivity is due to chance or systematic differences between the two cohorts.

The ASCO, NCCN and NBCG criteria include an assessment of the patient’s family history of cancer. The family histories of the mutation positive BC patients identified in our study were thoroughly investigated by genetic counselors and medical geneticists following the identification of the mutation, resulting in the sensitivity estimates presented. The observed estimates may therefore be higher than what is realistic in the clinical setting when family history is taken by the admitting physician at time of diagnosis. It may be difficult for the patient to know or recall detailed information about their family history of cancer when asked in a possibly stressful diagnostic setting. In line with this, Høberg-Vetti et al. found in their study from the Western part of Norway that 2 out of 26 (7.7%) mutation carriers reported a negative family history of cancer at time of diagnosis and testing, but closer evaluation revealed that they did have a family history of breast and/or ovarian cancer [32]. We also worry that the complexity of the NCCN, ASCO and NBCG criteria make them difficult to use and implement systematically in a busy clinical setting. Both these aspects could lead to fewer patients being offered testing, even those fulfilling the criteria. This is illustrated in several studies. Febbraro and colleagues observed that only 34% of breast cancer patients fulfilling NCCN guidelines were referred to genetic counseling and testing [51]. In a recent Swedish study where all BC patients were tested retrospectively, it was found that 65% of the mutation carriers fulfilled Swedish criteria for testing, but only 18% had been identified in regular clinical routine [52]. Moreover, even though all mutation carriers fulfilled the NCCN criteria in the study by Tung et al., 13.3% of the carriers identified through this research project had not been tested clinically [35].

The fact that 37% of the women had a family history of cancer that according to the Norwegian guidelines qualified for referral to predictive genetic testing before their own disease, may be another illustration of the challenges with using assessment of family history as a criteria for genetic testing or referral to genetic counseling. The low number leads us to conclude that the current system of referring healthy women to genetic testing based on their family history is suffering from lack of compliance. These women contracted cancers that could have been prevented had they known about their risk and undergone prophylactic surgery. The reasons for this lack of referral and how it can be improved need to be further explored, but this was not the scope of the current study.

Using age of onset as a criteria for testing will likely lead to increased adherence by surgeons and oncologists compared to guidelines requiring a detailed and complicated assessment of the patient’s family history of cancer. Testing all BC patients below 60 years identified as many or more carriers than all guidelines assessed (see Table 3). Due to the lowered cost of testing and the clinical impact of detecting a *BRCA* mutation, Finch et al. [53] have recently argued that the threshold for testing should be lowered from a 10% prior probability of being a carrier to 5%. Testing all under 60 in the OUH-U cohort gave a mutation detection rate of 5.5% (see Table 5), i.e. within this threshold. By using this criteria one would have to test 18 BC patients to identify one carrier. As of August 2016, testing these 18 patients had also led to the identification of one female relative per index patient. In Cohort 1 from OUH-U, 235 out of the 440 tested (53.4%) were younger than 60 and 132/440 (30%) fulfilled the old NBCG criteria (see Table 2). In 2014, 3324 Norwegian women contracted BC [54]. Using the calculations from the OUH-U cohort indicate that testing all below 60 years will involve 800 more analyses annually compared to testing only those fulfilling the old NBCG criteria.

One year after the last BC patient in our cohort was tested, 1.1 female relative per identified carrier had tested positive for the mutation and were given the opportunity of cancer prevention. It is likely that this number will increase as more relatives are informed and tested. According to Finch et al., “the value of a cancer genetic testing program comes from the number of cancers prevented” [53]. Even though testing all below 60 years may be feasible and effective, we observed that 10% will still be missed by this strategy. Two mutation carriers were older than 70 years. One may argue that the identification of a mutation in a woman who is 70 years or older may not influence treatment decisions, life expectancy or lead to a significant gain in quality adjusted life years (QALY) for this woman. However, it is likely that women over 70 have adult female relatives that may be at high risk of cancer due to the mutation.

We observed that more than half of the mutation carriers did *not* have a family history of breast and/or ovarian cancer before they were diagnosed with breast cancer themselves. These findings are in line with other studies reporting that family history has limited value in predicting carrier status [33, 38, 55], and our findings illustrate the difficulties with finding these women prior to disease development. Today, these women cannot obtain genetic testing while still healthy, as a population-based screening protocol is not accessible. Mary Claire King and colleagues consider that the identification of “a woman as a carrier only after she develops cancer is a failure of cancer prevention” [56] and based on their finding that *BRCA* mutation carriers have a high risk of cancer regardless of their family history [57], argue for population based screening to all women aged 30 years [56].

In a cost analysis of the cancer genetic services in the UK, Slade et al. have demonstrated that the most cost efficient genetic service model is to identify unaffected mutation carriers through an affected mutation positive index person [58], and argue for more comprehensive testing of all cancer patients fulfilling the NICE criteria. Patients fulfilling these criteria have an a priori 10% risk of being carriers. We identified a mutation in 3.1% of carriers, and one may argue that this is too low to warrant testing of all BC patients. We have however, recently shown that the practice of *BRCA* testing at OUH-U is cost-effective within the frequently used thresholds in Norway [59]. The cost-effectiveness was mainly due to the prevented breast-and ovarian cancers in their female relatives who tested positive for the mutation. Possible life years gained (LYG) due to prophylactic surgery among the BC patients was not included in the calculations in this study. The calculations may therefore be considered a conservative estimate. In addition, the cost of testing is constantly dropping, making the cost-effectiveness of a broad application of *BRCA* testing to BC patients even larger in the coming years.

Our results indicate that by testing only for founder mutations in the BC population of the South-Eastern part of Norway, and by testing only those with a family history of cancer, a significant number of mutation carriers will be missed. One may ask whether these results are relevant for screening strategies in other populations. The prevalence of *BRCA* mutations vary between populations [34, 60, 61], and the indication for genetic screening of all breast and ovarian cancer patients may be stronger in populations with a higher frequency of mutation carriers than in Norway. In populations where there is a stronger founder effect, the number of mutation carriers missed by offering testing for only founder mutations will be lower than what we have observed. However, recent studies have demonstrated that 13% of *BRCA1* mutations and 7.2% of *BRCA2* mutations in Ashkenazi Jews were non-founders [62]. Similarly, a Polish study found that in families with a family history of breast and/or ovarian cancer having tested negative for Polish *BRCA* founder mutations, sequencing revealed 31 other *BRCA* mutations. The detection rate of these mutations was 10% [63]. Sequencing and MLPA may therefore be warranted also in populations with a stronger founder effect than in Norway. We observed that only 40% of mutation carriers had a family history of breast and/or ovarian cancer. There are various reasons for this: Small family size, mutations may be inherited through several generations of men and incomplete penetrance. Family history as a selection tool for testing may have a higher sensitivity in populations with higher birth rates than in Norway. However, most western countries have had a declining birth rate since the 1960s and now have a birth rate between 1.5 and 2 [64]. One may therefore hypothesize that the value of using family history as a selection tool for testing will be even lower in the future.

BC patients are now often offered multi gene panel tests, and this is the direction in which the field of genetic testing is moving rapidly. There are several advantages with this strategy compared to testing only for the *BRCA* genes. More carriers of pathogenic mutations in other known BC risk genes such *TP53*, *PTEN* or *PALB2* will be identified. In addition, carriers of mutations in genes that likely would not have been investigated when testing only for one gene at a time will be identified. By testing a sequential series of breast cancer patients for 25 cancer predisposition genes, Tung and colleagues identified carriers of mutations in the *MSH6* and *PMS2* genes [35]. Conversely, by testing families suspected to have Lynch Syndrome for 112 known or candidate colorectal cancer genes, Hansen and colleagues identified one *BRCA1* carrier and two *BRCA2* carriers [65]. In sum, through multi gene panel testing more mutation carriers and their mutation positive relatives will be identified and given the opportunity of appropriate cancer surveillance and/or prevention. In the coming years this technology will also likely become more cost effective than traditional Sanger sequencing of one gene at a time. The aim of this study was not to argue against the value of multi gene panel testing, but rather to investigate whether the current strategies for *BRCA* testing, regardless of technology used, are sufficient to identify all carriers of mutations in these well-known and defined genes.

One limitation to our study is that we have not tested all BC patients. In the OUH-U cohort (Cohort 1) 167/607 = 27.5% of all women diagnosed with BC were not tested. These women were older and fewer filled the NBCG criteria than those who were tested. Unfortunately, we do not have access to the exact number of untested patients in the SERHA series or clinical information about these. If 2400 were treated in SERHA in the study period, about 39% of these (931/2400) were tested. The reason for the lower number of tested in Cohort 2 may be that there was a lower awareness of the possibility of genetic testing at these hospitals, but we cannot exclude that this cohort may be more selected. To assess this, we compared the two cohorts indirectly by comparing the mutation positive BC patients. There was a tendency towards a higher age of onset in Cohort 2 from SERHA, but this difference was not statistically significant. No significant differences were found between mutation carriers in the two cohorts in terms of TNBC and family history (Additional file 3: Table S2). We also observed the same frequency of mutation carries in the two series. The two cohorts may therefore be similar, and it is likely that the untested in Cohort 2 were older and that fewer filled the NBCG criteria than the tested. If there are mutation carriers among the untested in both series, the total frequency of carriers might have been lower, but it is likely that even fewer would have fulfilled the different high-risk criteria.

## Conclusions

By offering *BRCA* testing to a broad group of BC patients we found that 3.1% carried a deleterious mutation, and so far this has led to the identification of 1.1 female mutation positive relative per mutation positive BC patient. Even though mutation carriers differed as a group from mutation negative, criteria for testing based on the high-risk aspects did not detect all *BRCA* carriers in this BC population. Testing all BC patients below 60 years had a sensitivity matching the commonly used guidelines, and will likely be easier to apply, but 10% of mutation carriers would still be missed. Thirty-seven percent of the women had a family history of cancer prior to their own BC that qualified for predictive genetic testing. They contracted cancers that could have been prevented if the health care system had identified their increased genetic risk. Based on our combined observations, we conclude that the current strategies for *BRCA* testing are insufficient to detect all carriers. We suggest that it is time to discuss whether *BRCA* testing should be offered also to BC patients not belonging to a high risk group. If all BC patients are offered *BRCA* testing, the potential for cancer cure and prevention associated with such testing can be improved even further than what today’s strategies for testing allows. In case of lack of economic resources to fulfill this strategy, at least those aged 60 years or less at time of BC diagnosis should be tested. Our observations also indicate that health services need to be aware of referral possibilities for healthy women with cancer in the family, and the reasons for the low compliance should be explored. Improved strategies both for diagnostic and predictive *BRCA* testing will identify more mutation positive women prior to cancer development than the current practice.

## Additional files

Additional file 1: Figure S1. Guidelines for testing. (DOCX 26 kb)

Additional file 2: Table S1 Clinical and pathological characteristics of breast cancer in mutation carriers, non-carriers and not tested at OUH-U. (DOCX 28 kb)

Additional file 3: Table S2. Comparison of mutation positive breast cancer patients in Cohort 1 (OUH-U) and Cohort 2 (SERHA). (DOCX 14 kb)

## Abbreviations

ASCO: The American Society of Clinical Oncology
BC: Breast Cancer
CRN: Cancer Registry of Norway
DMG: Department of Medical Genetics
EPR: Electronic patient record
LYG: Life years gained
M: Distant metastasis
MLPA: Multi Ligation Probe Amplification
N: Involvement of regional lymph nodes
NBCG: The Norwegian Breast Cancer Group
NBCR: Norwegian Breast Cancer Registry
NCCN: The National Comprehensive Cancer Network
NICE: The National Institute for Health and Care Excellence
NNT: Numbers needed to test
NSD: Norwegian Social Science Data Services
OC: Ovarian cancer
OUH-U: Oslo University Hospital, Ullevål
REK: Regional Committees for Medical and Health Research Ethics
SERHA: South-Eastern Norway Regional Health Authority
T: Size of original tumor
TNBC: Triple negative breast cancer
TNM: Scoring of tumors according to the TNM Classification of Malignant Tumors
VUS: Variant of Uncertain Significance
