# Supplementary material for: Current guidelines for BRCA testing of breast cancer patients are insufficient to detect all mutation carriers
Source: BMC Cancer. 2017 Jun 21;17:438. doi: 10.1186/s12885-017-3422-2 (PMC5480128; doi:10.1186/s12885-017-3422-2)
Supplement: Supplementary file 1 — Guidelines for testing. (DOCX 26 kb) [file 12885_2017_3422_MOESM1_ESM.docx]

**Additional file 1: Figure S1: Guidelines for testing**

**1a: The National Comprehensive Cancer Network (NCCN) [24]**

**NCCN Guidelines Version 2.2017**

*BRCA1/2* testing criteria for breast cancer patients *

- Individual from a family with a known deleterious *BRCA1/BRCA2* gene mutation
- Personal history of breast cancer + one or more of the following:
  - Diagnosed ≤45 years
  - Diagnosed ≤50 years with:
    - An additional breast cancer primary
    - ≥1 close blood relative with breast cancer at any age
    - ≥1 close relative with pancreatic cancer
    - ≥1 relative with prostate cancer (Gleason score ≥7)
    - An unknown or limited family history
  - Diagnosed ≤60 years with:
    - Triple negative breast cancer
  - Diagnosed at any age with
    - ≥2 close blood relatives with breast cancer, pancreatic cancer, or prostate cancer (Gleason score ≥7) at any age
    - ≥1 close blood relative with breast cancer diagnosed ≤ 50 years
    - ≥1 close blood relative with ovarian carcinoma
    - A close male blood relative with breast cancer
    - For an individual of ethnicity associated with higher mutation frequency (eg, Ashkenazi Jewish) no additional family history may be required
  - Personal history of ovarian cancer
  - Personal history of male breast cancer
  - Personal history of prostate cancer with family history**
  - Personal history of pancreatic cancer with family history**
  - BRCA1/2 mutation detected by tumor profiling in the absence of germline mutation analysis**
  - First – or second-degree blood relative (same side of family) meeting any of the above criteria**
  - Third-degree blood relative who has breast/ovarian cancer and > 2 close blood relatives < 50 years or ovarian cancer**

*Breast cancer includes invasive carcinoma and DCIS

**Criteria not relevant for this study, for details on family history specifications please see the full NCCN guide.

**1b: American Society of Clinical Oncology (ASCO) [23]**

**ASCO Guidelines**

Breast Cancer Survivorship Care Guideline

“Those with a family history of breast or ovarian cancer or cancer in a certain age group and/or cancer type should be referred for genetic counseling for consideration of testing for hereditary predisposition to genetic mutations. Specifically, genetic counseling for consideration of testing for hereditary predisposition to gene mutations should be recommended for breast cancer survivors with the following characteristics:

1) those with at least one grandparent of Ashkenazi Jewish heritage,

2) younger than age 50 years at diagnosis,

3) with a history of ovarian cancer at any age or in any first-degree or second-degree relative,

4) with a first-degree relative who had breast cancer diagnosed before age 50 years,

5) with two or more first- degree or second-degree relatives diagnosed with breast cancer at any age,

6) with a diagnosis of bilateral breast cancer,

7) with a history of breast cancer in a male relative,

8) or any survivor diagnosed at age 60 years or younger with triple-negative breast cancer.

**1c: Norwegian Breast Cancer Group (NBCG) [26]**

**NBCG guidelines**

- Breast cancer <50 years
- Two close relatives with breast cancer, mean age <55 years
- Three close relatives with breast cancer at any age
- Male breast cancer
- Bilateral breast cancer <60 years
- Female breast cancer and close relative with ovarian cancer
- Female breast cancer and close relative with prostate cancer <55 years
- Ovarian cancer at any age
- Female breast cancer patients up to 60 years with triple negative breast cancer should be offered testing
