# Supplementary material for: Current guidelines for BRCA testing of breast cancer patients are insufficient to detect all mutation carriers
Source: BMC Cancer. 2017 Jun 21;17:438. doi: 10.1186/s12885-017-3422-2 (PMC5480128; doi:10.1186/s12885-017-3422-2)
Supplement: Supplementary file 2 — Clinical and pathological characteristics of breast cancer in mutation carriers, non-carriers and not tested at OUH-U. (DOCX 28 kb) [file 12885_2017_3422_MOESM2_ESM.docx]

|  | BRCA 1/2 carriers(*n*=13)(%) | Non-carriers(*n*=427) **(%)** | Not tested(*n*=167) **(%)** | ***p*-values:**  **mut/**  **normal** | **tested / not tested** | **mut/normal/not-tested** |
| --- | --- | --- | --- | --- | --- | --- |
| Age at diagnosis | (*n*=13) | (*n*=427) | (*n*=167) |  |  |  |
| Mean (95 % C.I) | 42 (36.1-47.9) | 57.9 (56.8-59.1) | 65.3 (63.6-67.1) | <0.001 | <0.001 | <0.001 |
| Median (range) | 43 (26-58) | 58 (23-93) | 65 (28-92) |  |  |  |
| NBCG criteria before diagnosis | 4 (30.8) | 49 (11.5) | 9 (5.4) | 0.04 | 0.02 | 0.004 |
| NBCG criteria including personal diagnosis | 12 (92.3) | 130 (30.4) | 12 (7.2) | <0.001 | <0.001 | <0.001 |
| **TNM** | (*n*=13) | (*n*=422) | (*n*=162) |  |  |  |
| T1 | 5 (38.5) | 261 (61.8) | 103 (63.6) | 0.4 | 0.9 | 0.8 |
| T2 | 5 (38.5) | 121 (28.7) | 42 (25.9) |  |  |  |
| T3 | 2 (15.3) | 26 (6.2) | 12 (7.4) |  |  |  |
| T4 | 1 (7.7) | 13 (3.1) | 5 (3.1) |  |  |  |
| N0  N1  N2  N3 | 8 (61.5)  2 (15.4)  3 (23.1)  0 (0.0) | 04 (72.0)  82 (19.4)  24 (5.7)  12 (2.8) | 113 (70.2)  35 (21.7)  12 (7.4)  1 (0.6) | 0.08 | 0.4 | 0.15 |
| Distant metastasis | 0 (0.0) | 2 (0.5) | 1 (0.6) | 0.8 | 0.3 | 0.6 |
| **Grade** | (*n*=13) | (*n*=419) | (*n*=161) |  |  |  |
| 1 | 0 (0) | 101 (24.1) | 45(27.9) | 0.001 | 0.005 | <0.001 |
| 2 | 4 (30.8) | 211 (50.4) | 93 (57.8) |  |  |  |
| 3 | 9 (69.2) | 107 (25.5) | 23 (14.3) |  |  |  |
| **Estrogen receptor-status** | (*n*=13) | (*n*=423) | (*n*=167) |  |  |  |
| Positive | 7 (53.8) | 371 (87.7) | 154 (92.2) | <0.001 | 0.06 | <0.001 |
| Negative | 6 (46.2) | 52 (12.3) | 13 (7.8) |  |  |  |
| **Progesterone receptor-status** | (*n*=13) | (*n*=422) | (*n*=167) |  |  |  |
| Positive | 5 (38.5) | 276 (65.4) | 124 (74.3) | 0.04 | 0.02 | 0.01 |
| Negative | 8 (61.5) | 146 (34.6) | 43 (25.7) |  |  |  |
| **HER2-status** | (*n*=13) | (*n*=423) | (*n*=167) |  |  |  |
| Positive | 2 (15.4) | 57 (13.5) | 10 (6.0) | 0.8 | 0.09 | 0.03 |
| Negative | 11 (84.6) | 366 (86.5) | 157 (94.0) |  |  |  |
| TNBC | 5 (38.5) | 30 (7.0) | 10 (6.0) | <0.001 | 0.4 | <0.001 |
| **Ki67** | (*n*=13) | (*n*=412) | (*n*=149) |  |  |  |
| Mean (95% (C.I)  > 30% activitiy | 59 (45.3-72.78)  11 (84.6) | 31.3 (29-33)  182 (44.1) | 25.5 (22–28.32)  50 (33.0) | <0.001 0.004 | 0.003  0.01 | 0.01  0.001 |
