# Supplementary material for: Current guidelines for BRCA testing of breast cancer patients are insufficient to detect all mutation carriers
Source: BMC Cancer. 2017 Jun 21;17:438. doi: 10.1186/s12885-017-3422-2 (PMC5480128; doi:10.1186/s12885-017-3422-2)
Supplement: Supplementary file 3 — Comparison of mutation positive breast cancer patients in Cohort 1 (OUH-U) and Cohort 2 (SERHA). (DOCX 14 kb) [file 12885_2017_3422_MOESM3_ESM.docx]

| **Criteria** | **Cohort 1 (OUH-U)**  **(*n* = 13)** | **Cohort 2 (SERHA)**  **(*n*= 29)** | ***p* - value** |
| --- | --- | --- | --- |
| Age (mean) | 42 years  (31.9 -  51.8) | 48 ( 37.2 - 58.7) | 0.09 |
| Diagnostic NBCG criteria fulfilled | 12/13 (92.3 %) | 24/29 (83 %) | 0.6 |
| Triple negative breast cancer | 5/13 (38.5 %) | 9/29 (31 %) | 0.7 |

**Additional file 3: Table S2****. Comparison of mutation positive breast cancer patients in Cohort 1 (OUH-U) and Cohort 2 (SERHA)**
